# Levels of physical dependence on tobacco among adolescent smokers in Cyprus

**DOI:** 10.1016/j.addbeh.2016.04.004

**Published:** 2016-09

**Authors:** Costas A. Christophi, Despina Pampaka, Martha Paisi, Solonas Ioannou, Joseph R. DiFranza

**Affiliations:** a Cyprus International Institute for Environmental and Public Health in association with Harvard School of Public Health, Cyprus University of Technology, Limassol, Cyprus; b Department of Environmental Health, Harvard School of Public Health, Boston, MA, USA; c Department of Family Medicine and Community Health, University of Massachusetts Medical School, Worcester, MA, USA

**Keywords:** Smoking, Physical dependence, Latency, Cyprus adolescents, Tobacco dependence, Addiction

## Abstract

**Purpose:**

The purpose of this study is to assess tobacco dependence among Cypriot adolescents and examine its association to cigarette consumption and attitudes towards smoking.

**Methods:**

The current study used cross-sectional data from the 2011 Cyprus Global Youth Tobacco Survey which adopted multistage cluster sampling methods to select adolescents registered in middle and high schools in Cyprus. Tobacco use, physical dependence on tobacco, and attitudes towards tobacco use were measured in 187 adolescents aged 13–18 years old who reported that they had smoked at least once in the preceding 30 days. Physical dependence was assessed using the Levels of Physical Dependence scale.

**Results:**

Physical dependence was present in 86% of the adolescent smokers. The mean latency to needing among smokers in the highest dependence group was 101 h. Significant associations were observed between physical dependence and the perceived difficulty in quitting (OR = 13.1, 95% CI: 4.0, 43.0) as well as the expectation to continue smoking for the next five years (OR = 3.3, 95% CI: 1.3, 8.4). Significant associations were also observed between physical dependence and the number of smoking days per month, daily smoking, daily cigarette consumption, lifetime cigarette consumption, and perceived difficulty in abstaining from smoking for one week.

**Conclusions:**

Physical dependence provides a symptom-based approach to assess dependence and it is a strong predictor of adolescents' perceptions of their ability to quit or to refrain from smoking for a week. Physical dependence on tobacco was highly prevalent among adolescent smokers in Cyprus and it was associated with greater perceived difficulty in quitting. Interventions targeting adolescent smoking must account for the high prevalence of physical dependence.

## Highlights

• Physical dependence on tobacco was present in 86% of the adolescent smokers.
• Withdrawal symptoms were not experienced until an average of 4 days of abstinence.
• Physical dependence was associated with greater perceived difficulty in quitting.
• Adolescent smokers in Cyprus had high levels of physical dependence.

## 1 Introduction

Smokers experience nicotine withdrawal symptoms when they abstain from smoking for a long time; this is a manifestation of physical tobacco dependence. Once dependence develops, smokers experience a physiologic need to smoke every time they go without smoking for some time (Ursprung, Morello, Gershenson, & DiFranza, 2011). Clinical studies established that physical dependence (PD) develops in well-defined stages that progress similarly in all smokers (DiFranza, Sweet, Savageau & Ursprung, 2011). These stages are *wanting*, *craving*, and *needing* and can be assessed using the Levels of Physical Dependence scale (DiFranza, Wellman, & Savageau, 2012). Scores on this scale correlate well with structural alterations in the addiction circuitry of the brain (Huang, W, et al., 2013, Huang, W, et al., 2014). Although these levels only assess PD, advancement through the different stages corresponds to higher daily and lifetime consumption, and overall addiction (DiFranza et al., 2012).

Smokers who developed PD can only forgo smoking for so long before they begin to experience a physiologically triggered desire to smoke. The time between the last cigarette and the onset of the desire to smoke is termed the *latency* (DiFranza & Ursprung, 2008). Depending on the level of PD that the smoker has reached, during withdrawal from nicotine the desire to smoke can intensify from wanting, to craving, to needing. Thus, there is latency to wanting, latency to craving, and latency to needing (Fernando, WWSA, et al., 2006, Ursprung, WW, et al., 2011). At the onset of PD, the latencies may be measured in weeks, but over time, as tolerance develops, they shorten progressively. This shortening drives an increase in tobacco consumption (Fernando, WWSA, et al., 2006, Ursprung, WW, et al., 2011).

It is argued that the earlier in age smoking is initiated the higher are the odds of becoming dependent (Breslau, Fenn, & Peterson, 1993). Even simple experimentation with tobacco by adolescents significantly raises their risk of being smokers as adults (Chassin, L, et al., 1996, McPherson, L, et al., 2008). Dependence symptoms present early in the process, driving consumption and leading to regular smoking (Fernando et al., 2006). Nicotine dependence may affect the switch from experimentation to becoming a regular tobacco user (Rojas, Killen, Haydel, & Robinson, 1998) and may escalate smoking frequency as this transition occurs (Rose & Dierker, 2010).

Social and environmental factors, such as tobacco advertising, and peer and parental smoking, are important factors for smoking initiation (Rojas et al., 1998), smoking prevalence (Christophi, Savvides, Warren, Demokritou, & Connolly, 2009), smoking dependence, and intent to quit (Savvides et al., 2014). A better understanding of how PD on tobacco develops in adolescents may be helpful for preventing tobacco use among youth.

Cyprus is an island in the eastern Mediterranean region with a population of 847,000 people (Statistical Service of the Republic of Cyprus). The Republic of Cyprus has five government-controlled municipalities, namely Lefkosia, Lemesos, Paphos, Larnaka, and Ammochostos. Despite the fact that adolescent smoking prevalence has declined in some countries, it has increased in others, such as Cyprus. According to the Eurobarometer, about one third of the adult population in Cyprus reported being current smokers (TNS Opinion and Social, 2012) and Cyprus has one of the highest cigarette consumption rates in Europe (Eriksen, Mackay, & Ross, 2012). Furthermore, smoking prevalence among adolescents in Cyprus is quite concerning, at 13% among boys and 7% among girls in middle-schools, and 36% among boys and 23% among girls in high-schools (Christophi et al., 2008). Between the 2006 and 2011 Global Youth Tobacco Survey (GYTS) in Cyprus, daily cigarette smoking increased, in relative terms, by 3.7%. This happened despite measures taken by the government of Cyprus, including smoking prevention programs within schools and a ban on smoking in all enclosed public spaces (Christophi et al., 2013).

As the levels of PD and latencies phenomena have only been studied in convenience samples, we sought to extend this work by studying a systematic sample of adolescent smokers in Cyprus. As this is the first such study in Cyprus, it is important to understand how prevalent PD is among Cypriot adolescents, and how PD influences attitudes and expectations about quitting. Understanding addiction is vital for developing effective intervention programs.

## 2 Methods

### 2.1 Study design

A two-stage cluster design was used to select a sample of adolescents registered in middle- (grades 7–9) and high- (grades 10–12) schools in Cyprus. Standardized methodology used in the GYTS was followed; details can be found elsewhere (GYTS Collaborating Group, 2003). Briefly, all middle and high schools with a school size of 40 or more students in the academic year 2009–2010 were included in the sampling frame; in the first stage schools were selected with probability proportional to their size and in the second stage classes within selected schools were chosen using a systematic equal probability sampling with a random start. All students in the selected classes were eligible to participate. The Cyprus Ministry of Education and Culture granted permission for the study and parental consents were obtained before students could participate. Participation was voluntary. The survey was administered in a class group setting in the students' classroom during school hours by trained field workers. The questionnaires were paper-based, self-administered in Greek, and completed anonymously. The overall response rate was 31% and the completed questionnaires were mailed to the US Centers for Disease Control for data entry. Because of the lower response rate, the questionnaires were not weighted for the analysis.

### 2.2 Measures

Our analysis focused on current smokers, defined as having smoked at least one cigarette during the past 30 days. All other participants were excluded from the analyses. Current smokers were further classified as daily and non-daily smokers.

The main variable of interest was tobacco PD as assessed by the Levels of Physical Dependence measure (DiFranza et al., 2012). This validated measure assigns smokers to one of four levels of PD based upon their endorsement of the following three statements:“*If I go too long without smoking the first thing I will notice is a mild desire to smoke that I can ignore*” (Level 1 PD: wanting); “*If I go too long without smoking*, *the desire for a cigarette becomes so strong that it is hard to ignore and it interrupts my thinking*” (Level 2 PD: craving); and “*If I go too long without smoking I just can*'*t function right*, *and I know I will have to smoke just to feel normal again*” (Level 3 PD: needing). Response options were “not at all, a little, pretty well, very well.” Using the standard scoring approach, endorsement of any of the three positive response options was considered to be an endorsement of the item. Participants were assigned a score from 0 to 3 reflecting the highest stage they had endorsed. Individuals who did not endorse any of the three statements were assigned a score of zero (Level 0 PD: no PD). The Levels of PD has been validated before both as a categorical and a continuous measure (DiFranza et al., 2012).

Other variables included: age, gender, number of smoking days/month (1–2, 3–5, 6–9, 10–19, 20–29, and every day), daily cigarette consumption (< 1, 1–5, 6–10, 11–20, and > 20 cigarettes), and lifetime cigarette consumption (< 10, 10–19, 20–99, 100 cigarettes or more). The mid-point of the reported range was used to create continuous variables.

Latencies were measured as follows; a) latency to wanting: “*How long can you usually go without smoking before you feel a mild desire to smoke*?”; *b*) latency to craving: “*How long can you usually go without smoking before you feel such a strong desire to smoke that it is hard to ignore*?”; and c) latency to needing: “*How long can you usually go without smoking before you feel you need to smoke just to feel normal again*?”*.* Participants were provided with response categories for hours, days, and weeks. Responses were converted to hours and treated as continuous variables in the analyses. In order for participants to have a latency to wanting they must have reached the wanting stage (Level 1 PD), to have a latency to craving-the craving stage (Level 2 PD), and to have a latency to needing-the needing stage (Level 3 PD).

Attitudes and expectations related to quitting were evaluated with the following: “*If you smoke now*, *have you thought about trying to stop smoking in the near future*?”, “*Do you think you will be smoking cigarettes 5 years from now*?”, “*How easy or difficult would you find it to go without smoking for as long as a week*?”, “*How easy or difficult would you find it to give up smoking altogether if you wanted to*?”, and “*Do you want to stop smoking now*?”, with the answers converted in dichotomous variables (‘yes/no’ or ‘difficult/easy’).

### 2.3 Statistical analysis

Qualitative variables were described using frequencies and percentages, while continuous variables using means and standard deviations. Groups were compared with the *t*-test or the Analysis of Variance technique for quantitative variables and the chi-square test for qualitative variables, or the Fisher's exact test, as appropriate. The Cochran-Armitage test was used to evaluate the trend across PD in relation to the attitudes considered. Correlation analysis was utilized to assess the strength of the linear association between PD, as a continuous variable, and other continuous characteristics. Multivariable linear and logistic regression techniques were used to model the association between the levels of PD and consumption and number of days smoked per month or the probability of being a daily smoker. Analyses were performed using SAS 9.3 (SAS Institute Inc., USA) and all tests reported are two-sided with any *p*-value of < 0.05 considered statistically significant.

## 3 Results

### 3.1 Sample characteristics

Questionnaires were completed by 1318 participants, 44 of whom were excluded due to missing data for smoking. Of the remaining 1274 participants, 217 (17.0%) were current smokers. Thirty of those had missing data on PD, leaving 187 individuals as the final sample for this report. The mean age in this sample was 15.7 ± 1.2 years with the majority of the participants being male (72%), having a lifetime consumption of 100 cigarettes or more (65%), with a mean daily consumption of 7.8 ± 7.8 cigarettes, and smoking on average 18 ± 12 days/month (Table 1).

### 3.2 Associations with PD

Participants were distributed across all levels of PD (Table 2); no PD (Level 0) was reported by 14.4% of current smokers, 17.1% were at Level 1 PD (wanting), 15.5% at Level 2 PD (craving), and 52.9% at Level 3 PD (needing). The proportion in Level 3 PD was higher than that in Level 2 PD (*p* < 0.001). There were no significant gender or age differences among the levels of PD (Table 2).

Higher levels of PD were associated with higher levels of daily consumption and more smoking days/month (Table 2, both *p* < 0.001). Smokers at Level 0 PD smoked an average of 3.35 cigarettes on smoking days and smoked an average of 7.2 days/month. In comparison, those at Level 3 PD smoked an average of 10.26 cigarettes on smoking days and smoked on average 21.8 days/month, indicating that youth could reach Level 3 PD even without being daily smokers. Higher levels of PD were also associated with higher lifetime consumption (*p* < 0.001). Among smokers at Level 1 PD, 13% had smoked fewer than 10 cigarettes in their lifetime and 53% had smoked 100 or more cigarettes, whereas at Level 2 PD and Level 3 PD, 67% and 79%, respectively, had a lifetime consumption of 100 or more cigarettes. Of the 118 subjects who had smoked 100 or more cigarettes, 65% were in fact at Level 3 PD.

While there was no significant difference in the mean Level of PD between boys and girls aged 11–14 years old (*p* = 0.42), among 15–17 year olds, girls had a greater mean dependence than boys (2.41 ± 0.95 vs. 1.98 ± 1.14, respectively, *p* = 0.029). Furthermore, participants with a lifetime consumption of at least 100 cigarettes had a higher mean Level of PD than those with fewer than 100 cigarettes (mean 2.4 ± 0.9 vs. 1.5 ± 1.2, respectively, *p* < 0.001). There was a moderate correlation between PD and both daily cigarette consumption (*r* = 0.37, *p* < 0.001) and the number of smoking days/month (*r* = 0.44, *p* < 0.001).

In a multiple linear regression model, adjusting for age and gender, Level 2 and Level 3 PD, but not Level 1, when compared to Level 0, were significant determinants of daily consumption with Level 3 being the strongest (Table 3). Similarly, when compared to Level 0, Level 2 and Level 3, but not Level 1, were significant determinants of the number of smoking days/month; age was also statistically significant (Table 3). In both unadjusted and adjusted for gender and age models, Level 2 and Level 3 PD were significant predictors of the probability of being a daily smoker (Level 2 adjusted OR = 11.4, 95% CI: 2.2, 59.3 and Level 3 adjusted OR = 19.6, 95% CI: 4.2, 91.0, Table 4).

### 3.3 Attitudes towards smoking

The mean PD was significantly higher among participants who believed they would be smoking in 5 years as compared to those who did not (2.2 ± 1.1 vs. 1.6 ± 1.3, respectively, *p* = 0.006). The proportion of smokers believing that they would be smoking 5 years from now increases with more advanced Levels of PD (Cochran-Armitage trend *p* = 0.006). Compared to participants at Level 0, participants at Levels 2 and 3 were more likely to believe that they would still be smoking in 5 years (Level 2 PD OR = 3.8, 95% CI: 1.1, 13.1 and Level 3 PD OR = 3.3, 95% CI: 1.3, 8.4, *p* = 0.037).

Students that found it difficult to go without smoking for a week had a higher mean PD as compared to those that found it easy (2.7 ± 0.7 vs. 1.4 ± 1.2, respectively, *p* < 0.001). The proportion of smokers perceiving refraining from smoking for a week difficult increases with more advanced Levels of PD (Cochran-Armitage trend *p* < 0.001). There were higher odds of finding it difficult to go without smoking for a week for smokers with Level 2 PD as compared to Level 0 (OR = 35.0, 95% CI: 3.9, 312.2) and for Level 3 PD compared to Level 0 (OR = 58.0, 95% CI: 7.3, 460.4).

Participants that found quitting difficult had a higher mean PD than those that considered it easy (2.5 ± 0.9 vs. 1.5 ± 1.2, respectively, *p* < 0.001). The proportion of smokers finding it difficult to give up smoking increased as the Level of PD increased (Cochran–Armitage trend *p* < 0.001). Higher Levels of PD were associated with a greater perceived difficulty of quitting (OR = 11.6, 95% CI: 2.9, 46.5, for Level 2 PD, and OR = 13.1, 95% CI: 4.0, 43.0, for Level 3 PD).

There were no significant associations between physical dependence and responses to the items “*If you smoke at present*, *have you thought about trying to stop smoking in the near future*?” or “*Do you want to stop smoking now*?”.

### 3.4 Latencies

As expected, the latency to wanting was shorter for participants at Level 2 or 3 PD, than Level 1 (Table 2). For 16 participants at Level 1 PD (wanting) the mean latency to wanting was almost 10 days (237 ± 125 h, Table 2). The latency to wanting did not correlate significantly with daily cigarette consumption (*r* = − 0.12, *p* = 0.65) or the number of smoking days/month (*r* = − 0.28, *p* = 0.30).

For 20 smokers in Level 2 PD (craving), the mean latency to craving was 85 ± 104 h (Table 2). Correlations between the latency to craving and daily consumption (*r* = − 0.43, *p* = 0.06) and number of smoking days (*r* = − 0.36, *p* = 0.11) were not statistically significant.

For 80 participants in Level 3 PD (needing), the mean latency to needing was 101 ± 117 h (Table 2). It correlated with daily cigarette consumption (*r* = − 0.37, *p* < 0.001) and with the number of smoking days/month (*r* = − 0.36, *p* = 0.001). The mean latency to needing was 165 ± 140 h (about 7 days) for adolescents who had smoked < 100 cigarettes compared to 90 ± 110 h (about 4 days) for those who had smoked 100 or more (*p* = 0.04).

## 4 Discussion

We compared the distribution of PD of our sample to convenience samples of American adults (ages 18–78, mean age 33) and adolescents (mean age 16) (DiFranza, JR, et al., 2012, DiFranza, J, et al., 2011a) (Table 5). The first observation is that PD is not normally distributed in these populations. This may reflect the fact that PD is not a normal physiologic condition; it is a progressive pathological condition that is more advanced in some individuals than others (Huang et al., 2013). The second observation is that more Cypriot than American adolescent (or adult) smokers have advanced to the highest Level of PD. We do not believe that this should be dismissed as a measurement error as the Level of PD scale correlates strongly with neural alterations in the brain (Huang, W, et al., 2013, Huang, W, et al., 2014). Also, the validity of the PD measure in our sample is supported by the significant associations between PD and cigarettes smoked per day, number of smoking days/month, lifetime consumption, perceived difficulty in abstaining for one week, perceived difficulty in quitting, and expectations to continue smoking for five years. The validity of the PD measure has been established in studies of adolescents in Spanish (Argentina), French (Canada), German (Germany), Arabic (Lebanon), and English (New Zealand and the United States). A conservative conclusion is that adolescent smokers in Cyprus may have more advanced PD than in other countries.

We did not observe a significant correlation between PD and motivation or intention to quit. Rather, we found that adolescents with more advanced PD were more likely to be pessimistic about their ability to quit compared to smokers who had not developed PD. Given the difficulty that adolescents have with smoking cessation (Sussman & Sun, 2009), this pessimistic assessment is probably realistic. Our study cannot address why Cypriot adolescents may be more vulnerable to developing advanced PD. One possibility is a shared genetic predisposition; another is a cultural environment that is more tolerant of tobacco use than other countries. Either explanation is consistent with the observation that adolescent smoking rates in Cyprus are among the highest in Europe.

Our data add to our knowledge about nicotine dependence in adolescents. Our results are consistent with prior studies indicating that PD often develops before the onset of daily smoking (O'Loughlin et al., 2003). Indeed, PD was a strong predictor of daily smoking (Table 4). Also consistent with prior studies is the observation that PD can develop with minimal lifetime tobacco exposures (Scragg, Wellman, Laugesen, & DiFranza, 2008). Significant individual differences in vulnerability to tobacco are evident: some individuals who had smoked fewer than 10 cigarettes in their lifetime were at the highest Level of PD, while others who had smoked 100 or more cigarettes had no PD. It should be noted that although the Level of PD measure correlates well with other measures of nicotine dependence, individuals at the highest Level of PD had a mean score of only 3.9 (out of 9) on the Modified Fagerström Tolerance Questionnaire (DiFranza, Sweet, et al., 2011). In contrast to all other measures of nicotine dependence, the Level of PD measures progression along a defined pathophysiologic process and does not rely on an assessment of behavior.

Intuition suggests that consumption may be a good indicator of addiction level. Indeed, our data show that with increasing Levels of PD there is an increase in both smoking days/month and daily consumption (Table 2). Yet some individuals with Level 1 PD smoke more than individuals at Level 3, so it is not possible to predict the severity of dependence based on the amount or frequency of tobacco use.

Among adult smokers, the latency to needing is typically much < 24 h (DiFranza, Morello & Gershenson, 2011). In our adolescents, the latency to needing was about 4 days. Longer latencies in adolescents than adults is consistent with the fact that the latencies shorten over time (Ursprung et al., 2011). Our data showing an inverse correlation between the latency to needing and both daily cigarette consumption (*r* = − 0.37, *p* < 0.001) and the number of smoking days/month (*r* = − 0.36, *p* = 0.001) supports the conclusion that a shortening of the latency to needing contributes to an escalation in smoking frequency during adolescence (DiFranza, 2015).

Study limitations include the relatively small sample that might have decreased our power to detect significant associations in some analyses; hence, our findings should be replicated in larger samples. In addition, the study had a low response rate but we do not believe that any bias was introduced because of this. Among the strengths of the study was the use of a sample of adolescent smokers in Cyprus, a nation with a high smoking rate. In addition, this was the first study using the Levels of PD in the GYTS and the first comparing it to attitudes and beliefs about quitting, which provided new evidence supporting the concurrent validity of this measure. Furthermore, this study extends the use of the Levels of PD to a different language and cultural setting.

## 5 Conclusions

Cultural differences regarding the price of tobacco and opportunities to smoke may bias comparisons between populations using behavior-based measures of dependence. PD levels provide a symptom-based approach to assess dependence that avoids this problem. The validity of the Level of PD measure was supported by its associations with other measures. PD is a particularly strong predictor of adolescents' perceptions of their ability to quit or to refrain from smoking for a week. In addition to having a high prevalence of smoking, Cypriot adolescent smokers may have more advanced PD than in other nations. The Levels of PD measure should be incorporated into the GYTS to allow for comparisons of PD across populations of adolescent smokers.

## Abbreviations

PD: physical dependence
GYTS: Global Youth Tobacco Survey
OR: odds ratio
CI: confidence interval

## Role of funding sources

The study was supported by the World Health Organization. The sponsor had no role in the study design, collection, analysis or interpretation of the data, writing the manuscript, or the decision to submit the paper for publication.

## Contributors

JRD and CAC designed the study and DP, SI, and CAC performed the statistical analysis. CAC developed the first draft of the manuscript with DP, MP, and SI. JRP, DP, and MP critically revised an earlier draft of the manuscript and all authors read and approved the final manuscript.

## Conflict of interest

The authors declare that they have no conflicts of interest.

## Figures and Tables

**Table 1:** Sample characteristics.

	N (%)
Gender	
Males	135 (72%)
Females	52 (28%)
Lifetime cigarette consumption	
< 10	17 (9%)
10–19	14 (7%)
20–99	34 (19%)
100 or more	118 (65%)
If you smoke at present, have you thought about trying to stop smoking in the near future? (% yes)	106 (72%)
Do you think you will be smoking cigarettes 5 years from now? (% yes)	130 (75%)
How easy or difficult would you find it to go without smoking for as long as a week? (% easy)	68 (47%)
How easy or difficult would you find it to give up smoking altogether if you wanted to? (% easy)	61 (40%)
Do you want to stop smoking now? (% yes)	55 (42%)
	Mean ± SD
Age (years)	15.7 ± 1.2
Daily cigarette consumption	7.8 ± 7.8
Days smoked in a month	17.9 ± 12.2

**Table 2:** Comparison of demographic and smoking variables across Levels of Physical Dependence.

	Level 0 PD No physical dependence	Level 1 PD Wanting	Level 2 PD Craving	Level 3 PD Needing	*p*-Value⁎
	*N* = 27 (14%)	*N* = 32 (17%)	*N* = 29 (16%)	*N* = 99 (53%)	
Gender					0.33
Males	85%	75%	72%	68%	
Females	15%	25%	28%	32%	
Age (years)	15.5 ± 1.3	15.8 ± 1.1	15.7 ± 1.2	15.6 ± 1.2	0.88
Lifetime cigarette consumption					< 0.001
< 10	19%	13%	4%	7%	
10–19	23%	6%	11%	3%	
20–99	35%	28%	18%	11%	
100 or more	23%	53%	67%	79%	
Daily cigarette consumption	3.35 ± 5.61	4.06 ± 4.83	7.98 ± 7.26	10.26 ± 8.23	< 0.001
Days smoked in a month	7.2 ± 8.6	12.6 ± 10.9	20.1 ± 11.8	21.8 ± 11.2	< 0.001
Latency to wanting (hours)	–	237 ± 125	88 ± 116	101 ± 123	< 0.001
Latency to craving (hours)	–	–	85 ± 104	97 ± 116	0.68
Latency to needing (hours)	–	–	–	101 ± 117	–

⁎ Testing for difference among the 4 groups of PD (Chi-square or ANOVA test).

**Table 3:** Linear regression models examining Level of Physical Dependence as predictors of daily cigarette consumption and number of smoking days in a month.

	Daily cigarette consumption		Number of smoking days in a month	
	Coefficient	*p*	Coefficient	*p*
Level 1 PD vs. Level 0	0.74	0.71	5.11	0.07
Level 2 PD vs. Level 0	4.65	0.02	12.75	< 0.001
Level 3 PD vs. Level 0	7.08	< 0.001	14.86	< 0.001
Age (year)	0.82	0.08	2.47	< 0.001
Gender (male vs. female)	0.79	0.52	0.91	0.61

**Table 4:** Logistic regression models examining Level of Physical Dependence as a predictor of daily smoking.

Effect	OR	Lower 95% CI	Upper 95% CI
*Model 1 — Unadjusted*			
Level 1 PD vs. Level 0	2.32	0.41	13.03
Level 2 PD vs. Level 0	11.67	2.32	58.59
Level 3 PD vs. Level 0	18.44	4.13	82.22

*Model 2 — Adjusted for gender and age*			
Level 1 PD vs. Level 0	2.09	0.36	12.10
Level 2 PD vs. Level 0	11.38	2.18	59.32
Level 3 PD vs. Level 0	19.61	4.23	90.96
Gender (female vs. male)	0.93	0.44	1.96
Age (year)	1.58	1.18	2.13

**Table 5:** Comparison of the distribution of individuals across the Levels of Physical Dependence in three studies.

Level of physical dependence	Level 0 None	Level 1 Wanting	Level 2 Craving	Level 3 Needing
Cypriot adolescents (*n* = 187)	14%	17%	16%	53%
American adolescents (*n* = 347)^a^	48%	26%	6%	20%
American adults (*n* = 422)^b^	17%	26%	17%	40%

a DiFranza, Sweet, et al. (2011).

b DiFranza et al. (2012).
